# Role of tRNA-Derived Fragments in Protozoan Parasite Biology

**DOI:** 10.3390/cells14020115

**Published:** 2025-01-14

**Authors:** Manu Sharma, Upinder Singh

**Affiliations:** 1 Division of Infectious Diseases, School of Medicine, Stanford University, Stanford, CA 94305, USA; msharma4@stanford.edu; 2 Department of Microbiology and Immunology, School of Medicine, Stanford University, Stanford, CA 94305, USA

**Keywords:** tRNA fragments, protozoan parasite, extracellular vesicles, small RNA, tsRNA

## Abstract

**Highlights:**

- tsRNAs are small RNAs generated through specific cleavage of parent tRNAs, found across all domains of life, including protozoan parasites.
- tsRNAs are classified into tRNA halves formed by cleavage of a mature tRNA molecule at the anti-codon loop, or tRNA fragments, formed by cleavage of both pre- and mature- tRNAs at various positions.
- Various functions of tsRNAS have been identified including modulation of gene expression, translation inhibition, and response to cellular stress.
- tsRNAs have also been found to be packaged into extracellular vesicles and transported into host cells to modulate their gene expression.
- tRFs are formed by cleaving parent tRNA molecules into halves or smaller fragments.

**Abstract:**

tRNA molecules are among the most fundamental and evolutionarily conserved RNA types, primarily facilitating the translation of genetic information from mRNA into proteins. Beyond their canonical role as adaptor molecules during protein synthesis, tRNAs have evolved to perform additional functions. One such non-canonical role for tRNAs is through the generation of tRNA-derived fragments via specific cleavage processes. These tRNA-derived small RNAs (tsRNAs) are present across all three domains of life, including in protozoan parasites. They are formed through the cleavage of the parent tRNA molecules at different sites, resulting in either tRNA halves or smaller fragments. The precise mechanisms underlying the synthesis of various tRNA-derived fragments, including the specific RNases involved, as well as their distinct functions and roles in parasite physiology, are not yet fully understood and remain an active area of ongoing research. However, their role in modulating gene expression, particularly during stress responses, is becoming increasingly evident. In this context, we discuss recent findings on the roles of tRNA-derived small RNA in various protozoan parasites. Furthermore, we investigate how these tsRNAs either modulate gene expression within the parasite itself or are packaged into extracellular vesicles to alter host gene expression, thereby promoting parasite survival and adaptation.

## 1. Introduction

All living organisms contain tRNA molecules, making tRNA one of the most fundamental and classical RNA types found in nature. In fact, it has been argued that the genetic code evolved around the tRNA and tRNA anticodon [1]. The first paper credited with the discovery of tRNAs as “soluble ribonucleic acid intermediates in protein synthesis” was published in 1958 [2]. Since then, tRNAs have been found to play a pivotal role in translating the genetic code from mRNA into proteins in all cellular life. However, during their long existence, tRNA evolved other functions besides their role as adaptor molecules during protein synthesis. It was not surprising, therefore, that later research revealed various non-canonical functions of tRNAs either directly or through generation of tRNA derived fragments through specific cleavage mechanisms (reviewed in [3,4]).

tRNA-derived small RNAs (tsRNAs) have been found to be present in all three domains of life [5]. tRNAs can be cleaved into noncoding RNAs (ncRNAs) particularly under stress conditions [6,7,8,9]. These tRNA-derived ncRNAs can be categorized into two main groups: tRNA halves, sometimes termed “tRNA-derived stress-inducible RNAs” (tiRNAs), and tRNA-derived fragments (tRFs).

tRNA halves consist of 30–35 nucleotides from either the 5′ or 3′ ends of mature tRNAs, formed through cleavage in the anticodon loop of mature tRNA [10]. In humans and other vertebrates, tRNA halves are generated by angiogenin, an enzyme belonging to the RNase A family upon exposure to stress [8,10]. Under normal conditions, angiogenin is bound to its inhibitor, Rnh1. During stress, angiogenin is released from Rnh1 and proceeds to cleave tRNA. However, angiogenin independent synthesis of tRNA halves has also been reported in humans [11] and in other systems [12]. Protozoan parasites like *Trypanosoma brucei* and *Entamoeba histolytica* have no known homologs of angiogenin, and it is interesting that diverse mechanisms of tRNA half generation exist in these organisms though they appear to perform similar roles in response to stress [13,14].

**Figure 1:**
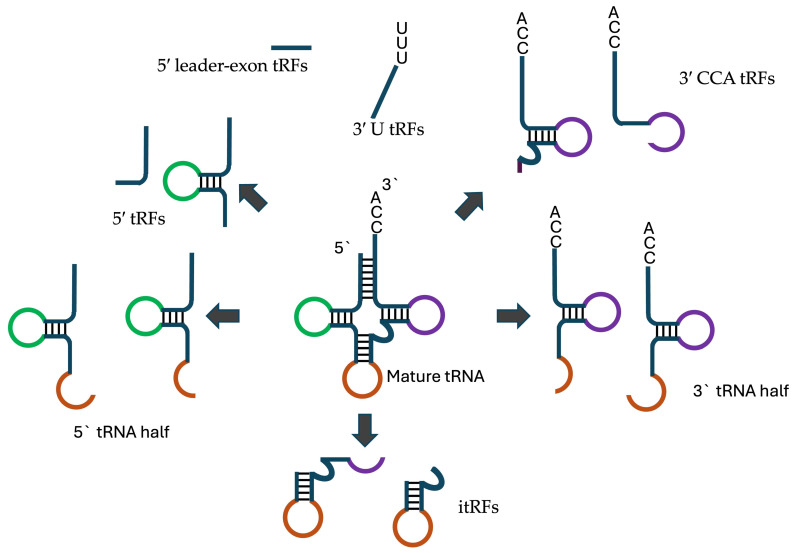
Various tRNA derived fragments.

tRNA-derived fragments are formed by cleavage of both pre- and mature- tRNAs at various positions to produce tRFs between 13 and 32 nucleotides in length Figure 1. Based on their site of origin, tRFs are classified into several types [15]:

- 5′ tRFs: Derived from the 5′ end of mature tRNAs, usually involving cleavage at the D loop.
- 3′ CCA tRFs: Formed from the 3′ end of mature tRNAs, including the CCA tail, by cleavage at the T loop through Dicer, angiogenin, or other RNase A family members.
- 3′ U tRFs: Generated from the 3′ leader sequence of precursor tRNAs (pre-tRNAs), typically cleaved by RNaseZ at the U-rich region created during RNA polymerase III termination.
- Internal tRFs (itRFs): Result from cleavage at both the anticodon loop and either the D loop or TΨC loop.
- 5′ leader-exon tRFs: Contain the 5′ leader sequence of pre-tRNAs along with the 5′ part of mature tRNAs.

Here we summarize the findings from recent publications about tRNA derived fragments in different protozoan parasites.

## 2. *Entamoeba histolytica*

*Entamoeba histolytica* is the causative agent of amebiasis, a disease that can range from causing diarrhea to more severe symptoms such as abscess formation in the lungs, liver, or brain. Amebiasis is transmitted through the ingestion of cysts from fecal-contaminated food and water. These cysts transform into trophozoites in the terminal ileum, where they reproduce and parasitize the large intestine. Eventually, the trophozoites undergo encystation within the colon and the cysts are excreted via feces [16].

The amoeba genome contains a very high number of tRNA genes, with around 4500 copies [17,18]. These genes are arranged in unique tandem repeats, forming arrays that constitute over 10% of the genome. The 25 distinct array units each contain up to 5 tRNA genes, with some also encoding 5S RNA. While the exact function of these arrays remains unclear these could have a function in mediating trans inactivation or have a structural role in the parasite genome compensating for the absence of classical telomeres [17,19]. Irmer et al. showed that *E. histolytica* Glu^(TTC)^ and Tyr^(GTA)^ tRNA genes are required to mediate trans inactivation of the amoebapore-A gene achieved by episomal transfection of trophozoites with the plasmid psAP1 [20,21].

More recently, Sharma et al. reported the presence of various tRNA-derived fragments in *Entamoeba* through bioinformatic analysis of small RNA libraries [14]. tRNA halves were found to be the most abundant of the tRNA derived fragments. Most of the tRNA halves (around 80%) originated from just four tRNAs (tRNA^Ala_AGC^, tRNA^Ala_TGC^, tRNA^Asp_GTC^, and tRNA^Arg_TCT^). Despite this, tRF abundance did not correlate with either codon usage, or tRNA copy numbers, suggesting a complex relationship between tRNA fragmentation and gene expression. tRNA halves accumulated in the parasites in response to various stress stimuli, such as oxidative stress, heat shock, and serum starvation. Various tRNA halves were also observed when the trophozoites were subjected to the encystation process- which was not surprising since this process also involves stress on the parasites. Interestingly, the stress response was not mediated by a few specific tRNA halves, as multiple tRNAs appeared to be processed during the different stress conditions, similar to what is observed in *Giardia* during stress.

The accumulation of tRNA halves seemed to coincide with changes in protein translation levels. Specifically, during oxidative stress, there was a reduction in protein translation, while during heat shock, protein translation increased. The exact mechanism by which tRNA halves influenced these changes was not fully elucidated, but the data indicated that their accumulation might play a role in modulating the translation machinery under stress conditions. Blocking the accumulation of tRNA halves using leucinol led to changes in protein translation, further supporting the idea that tRNA halves were involved in regulating protein synthesis during stress.

Furthermore, the researchers identified some tRNA-derived fragments that were associated with *Entamoeba* Argonaute proteins, EhAgo2-2 and EhAgo2-3, which had a preference for different tRNA-derived fragment species. Finally, they demonstrated that tRNA halves were packaged inside extracellular vesicles secreted by the amoebas. The ubiquitous presence of tRNA-derived fragments, their association with the Argonaute proteins, and the accumulation of tRNA halves during various stress conditions, including encystation, suggested a nuanced level of gene expression regulation mediated by different tRNA-derived fragments in *Entamoeba* [14].

## 3. *Giardia*

Giardiasis is an enteric infection caused by the protozoan parasite *Giardia duodenalis* (also known as *Giardia lamblia* or *Giardia intestinalis*). The infection is transmitted through the fecal-oral route or through direct person-to-person contact, and is initiated through the ingestion of cysts, which colonize the intestine as trophozoites [22]. These trophozoites attach to the epithelial cells of the small intestine using adhesive disks. Giardia has undergone reductive evolution and therefore lacks typical cellular organelles such as an endosomal/lysosomal system, Golgi complex, peroxisomes, and mitochondria [23]. A network of peripheral vesicles (PVs) are used by Giardia to perform various functions, including the release of extracellular vesicles. Giardiasis remains the most prevalent protozoal enteric infection globally, particularly in areas with poor hygiene and sanitation practices [24].

One of the earliest studies in parasites to identify tRNA derived fragments was done on *Giardia lamblia* [25]. Li et al. discovered a novel class of small RNAs about 46 nucleotides long, originating from the 3′ end of mature tRNAs which they called stress-induced tRNAs (si-tRNAs). These RNAs were found to accumulate during encystation and in response to various stresses such as temperature shock and serum starvation. The si-tRNAs retained the 3′ CCA tail and were derived from the 3′ portion of fully matured tRNAs by cleavage site of the anticodon left arm. The study revealed that si-tRNAs were produced across the entire tRNA family in response to stress, and not just limited to a few specific tRNAs [25].

In a subsequent study by the same group, Jian-You Liao et al. found two major types of small RNAs, viz. endogenous siRNAs and tRNA-derived sRNAs, in the genome of *G. lamblia*. They showed that tRNA cleavage leads to the production of six distinct types of tRNA-derived sRNAs and this plays a crucial role in the differentiation process of *G. lamblia* [26]. Notably, despite the upregulation of the tRNA derived sRNAs during differentiation, the expression levels of the various tRNAs themselves remained stable throughout the process, suggesting that the production of tRNA-derived sRNAs was tightly controlled.

Natali et al. analyzed the RNA content of exosomal-like vesicles (ElVs) produced by different assemblages of *Giardia lamblia*—A, B, and E [27]. The analysis revealed that each assemblage′s ElVs contained distinct small RNA (sRNA) types, including ribosomal-small RNAs (rsRNAs), messenger-small RNAs (msRNAs), and transfer-small RNAs (tsRNAs), with a notable predominance of tsRNA-Gly, tsRNA-Gln, and tsRNA-Arg in assemblages A, B, and E, respectively. The study showed that ElVs contained a specific cargo of sRNAs and that the ElVs can be internalized by Giardia trophozoites. However, any specific biological functions could not be attributed to the tsRNA fragments in these vesicles.

In a similar study, Siddiq et al. found a significant presence of ribosomal RNA (rRNA)- and transfer RNA (tRNA)-derived small RNAs, along with short-interfering RNAs (siRNAs) and microRNAs (miRNAs), within the extracellular vesicles (EVs) of *Giardia* [28]. Interestingly, the authors showed that *Giardia* EVs could interact with bacterial membranes, enhancing their swimming motility but inhibiting bacterial growth, and biofilm formation. Furthermore, the RNA cargo in the EVs was required for this trans-kingdom communication.

## 4. *Trypanosoma cruzi*

Trypanosoma cruzi, an obligate intracellular parasite, is the causative agent of Chagas disease (CD), also known as American trypanosomiasis [29].

Garcia-Silva et al. first reported the presence of a highly abundant class of tRNA-derived small RNAs, predominantly originating from the 5′ halves of mature tRNAs in the size-fractionated cDNA library (20–35 nt) constructed from the epimastigote forms of *Trypanosoma cruzi* [30]. These tRNA halves, represented about 25% of the small RNA population and their expression was increased during nutritional stress. Interestingly, over 98% of the tRNA halves were derived from the 5′ halves of tRNA^Asp_GUC^, tRNA^Glu_CUC^ and tRNA^Glu_UUC^. The highly homogeneous population of 5′ tRNA halves, predominantly 30 nucleotides in length, indicated a highly regulated tRNA processing mechanism. Subcellular localization studies using FISH revealed that tRNA halves are recruited to specific granular structures within the cytoplasm. Interestingly, the 5′- and 3′-derived halves were present in different granules. In subsequent studies, they found that in nutrient-starved epimastigotes, tsRNAs colocalized with the TcPIWI-tryp protein, a unique Argonaute protein in trypanosomatids, and were recruited to specific cytoplasmic granules [31]. Further analysis using electron microscopy revealed that tsRNAs and TcPIWI-tryp proteins were primarily localized to reservosomes and other vesicular structures, including endosome-like vesicles and Golgi-like structures. This suggests that tsRNA biogenesis in T. cruzi is likely linked to endocytic/exocytic pathways. It was therefore not surprising to find tsRNA in the extracellular vesicles that were secreted during nutrient deprivation, and even taken up by mammalian cells [31]. To determine if the extracellular vesicles were capable of modulating gene expression in mammalian cells, Garcia-Silva et al. treated HeLa cells with extracellular vesicles from *Trypanosoma cruzi* [32]. Microarray assays revealed that a large set of genes in HeLa cells were differentially expressed following the incorporation of *T. cruzi*-derived EVs. The response primarily affected pathways related to the host cell cytoskeleton, extracellular matrix, and immune responses. Finally, the authors transfected the HeLa cells with labeled synthetic RNAs corresponding to the most abundant tRNA-derived small RNAs present in the extracellular vesicles. The transfection efficiency was checked with immunofluorescence and found to be over 90%. They found that tsRNA^Thr^ caused significant changes in the levels of 20 genes, while tsRNA^Leu^ only affected 3 genes. Some of the genes altered in the transfected cells were also affected by *Trypanosoma cruzi*-derived EVs. However, the effect of the tsRNAs on these genes was often opposite to the effect observed when the cells were treated with EVs, which suggests that although the tsRNA are capable of modulating the expression levels in the transfected HeLa cells, the EVs carry a multitude of other factors that result in the epigenetic changes seen. It was not possible to ascertain if the observed changes in gene expression result from a direct interaction between tsRNA and mRNA in the host cells or if they are “secondary” effects mediated by other factors within the host cells [32].

## 5. *Trypanosoma brucei*

The protozoan parasite *Trypanosoma brucei* is the causative agent for human African trypanosomiasis (HAT), commonly referred to as sleeping sickness. This parasite is transmitted through the bite of infected tsetse fly, predominantly affecting rural communities in sub-Saharan Africa. During transmission, the fly injects metacyclic-form parasites into the host′s skin, which then spread into the bloodstream as long, slender replicative forms [33]. During the first stage of the disease, the parasites are primarily confined to the host′s blood and lymphatic system. If left untreated, the disease advances to the second stage, where the parasites cross the blood-brain barrier and invade the central nervous system [34].

Even though *T. brucei* faces varying environmental conditions throughout its lifecycle, it largely lacks mechanisms to regulate the transcription of protein-coding genes. In an elegant study, Fricker et al. investigated the small non-coding RNA (ncRNA) interactome of ribosomes from *T. brucei* by sequencing small RNAs that co-purified with cytosolic ribosomes [13]. tRNA halves, predominantly mapping to the 5′ end of tRNAs, were found to be amongst the most abundant ncRNA. Moreover, the tRNA halves were strongly upregulated during stress conditions, and in the stationary phase compared to the exponential phase, although the degree of upregulation varied among the different tRNA halves. Three tRNA halves were found to be the most abundant ribosome-associated tRNA fragments during nutrient deprivation- 3′ RNA^Thr^ half and 5′ halves mapping to tRNA^Ala^ and tRNA^Asp^. In contrast to the 3′ RNA^Thr^ half, the level of 5′ tRNA^Ala^ decreased during stress suggesting that a tRNA-specific mechanism is involved. Of these 3 tRNA fragments, only 3′ RNA^Thr^ was seen to stimulate in vitro protein synthesis during stress recovery. Moreover, transfection with anti-sense oligonucleotides against endogenous tRNA^Thr^ 3′ half resulted in inhibition of translation during stress recovery. Mechanistic insights were gained when it was shown that 3′ RNA^Thr^ stimulates translation by facilitating mRNA binding to the ribosome. The effect was also found to be dependent on the removal of 3′ CCA end seen in almost all *T. brucei* tRNA during starvation and 3′ tRNA^Thr^ halves containing the CCA tail were unable to stimulate translation [13]. In a separate study, the 3′ CCA shortening of tRNAs in *T. brucei* exposed to nutritional stress was shown. This trimming of the 3′ CCA-tail left the tRNAs unsuitable substrates for translation. During recovery, tRNAs were repaired by CCA-adding enzyme and turned-on the translation machinery [35].

In subsequent work from this group, Brogli et al. discovered that during nutritional stress the 37-nucleotide 3′-tRNA^Thr^ half lacking the CCA tail is generated within the mitochondria [36]. During stress recovery this 3′-tRNA^Thr^ half interacts with mitochondrial ribosomes, stimulating protein synthesis thus enhancing mitochondrial function and energy production capacity. Interestingly, mitochondrial lysates were found to be capable of generating 3′ tRNA^Thr^ halves from total RNA samples, confirming that the enzyme for the tRNA cleavage was present inside the mitochondria. However, extensive experiments with RNAi against 33 candidates failed to identify the mitochondrial RNase.

Future research could identify the role of other tRNA fragments in the stressed cells to understand their individual as well as cumulative effect. The RNAase responsible for tRNA cleavage is yet to be determined and elucidating its identity and regulation could provide insights into the mechanisms driving tRNA processing under stress.

## 6. *Trichomonas vaginalis*

*Trichomonas vaginalis* is the causative agent of trichomoniasis, the most common non-viral sexually transmitted infection in the world [37]. In the US alone, more than 1 million people are infected with *T. vaginalis* each year [38]. The infection is linked to various negative sexual and reproductive health outcomes, including adverse birth outcomes, increased risk of HIV and other STIs, pelvic inflammatory disease (PID), infertility, and cervical cancer [39,40].

Wang et al. carried out deep sequencing of small RNA transcriptome from *Trichomonas vaginals* trophozoites to identify microRNAs and tRNA-derived small RNAs (tsRNAs). tRFs and tRNA-halves were found to map to the 3′-, 5′-, and middle part of tRNAs [41]. Interestingly, tRNA-derived small RNAs, mostly 5′ tRNA halves, were found to be the main type of small RNA in the extracellular vesicles secreted by *T. vaginalis* [42]. The abundance of the tRNA fragments did not correlate with the codon usage of their cognate tRNA of origin, which shows that these were not generated through indiscriminate cleavage of the parent tRNA. The EVs were found to be internalized in human cells by lipid raft-dependent endocytosis suggesting a possible mechanism of host gene expression modulation through these tRNA halves [42].

## 7. *Leishmania*

The obligate intracellular parasite, *Leishmania*, is responsible for causing Leishmaniasis. The kinetoplastid, *Leishmania*, alternates between two hosts: a mammalian host and a sandfly vector. It exists in two life stages: the promastigote (motile form) in the gut of the sandfly and the amastigote (non-motile form) within the macrophages of the vertebrate host [43].

Lambertz et al. analysed the small RNA cargo of EVs secreted by *L. donovani* and *L. braziliensis* and found a significant number of reads in both that mapped to tRNA genes. For both libraries, tRFs derived from tRNA^Asp^, tRNA^Gln^, tRNA^Glu^, and tRNA^Leu^ were the most abundant. No correlation was found between the predicted cellular amino acid usage and the relative expression of tRFs in these libraries, confirming that the tRF generation was not due to random cleavage of the tRNAs. Both 3′- and 5′- end tRFs and tRNA halves were identified, though 5′tRNA halves constituted the majority of the identified reads in both *L. donovani* and *L. braziliensis*. The tRF profile in Leishmania whole cells remains to be analyzed and it would be interesting to see how it changes during the different stages of the parasite’s lifecycle, and whether these tRFs play a functional role in regulating parasite gene expression.

## 8. *Toxoplasma*

*Toxoplasma gondii* is an obligate intracellular protozoan parasite capable of infecting nearly all warm-blooded animals, including humans, leading to zoonotic toxoplasmosis. The zoonotic disease has significant economic burden worldwide. The polyxenous protozoan has developed multiple transmission routes across different host species and transmission can occur through three different life-cycle stages: ingestion of infectious oocysts, ingesting tissue cysts, or ingestion of tachyzoites present in meat or blood products [44].

Galizi et al. provided the first experimental evidence for a tRNA cleavage pathway in apicomplexan parasites when they showed the cleavage in the anti-codon loops to yield both 5′ and 3′ tRNA halves in *Toxoplasma gondii* [45]. The tRNA half generation was enhanced during stress, and found to originate either from pre-tRNAs prior to the addition of the CCA sequence, or from mature tRNAs where the CCA tail was removed before the tRNA was cleaved at the anticodon loop. Interestingly, avirulent strains of *T. gondii* showed higher amounts of tRNA halves, as did metabolically quiescent bradyzoite and sporozoite stages, compared to the fast-growing tachyzoite [45].

## 9. *Plasmodium*

The apicomplexan protozoan *Plasmodium* species cause malaria, one of the most significant infectious diseases worldwide, particularly impacting children. The extent of the disease’s impact on humans can be gauged by the negative pressure on the human population during evolution, resulting in prevalence of genetic disorders like sickle cell anemia in malaria endemic regions [46]. The most serious infection is caused by *P. falciparum* with a wide range of pathologies, including severe anemia and cerebral malaria leading to death. The obligate intracellular parasite has a complex life cycle where sporozoites injected into the skin by bite of *Anopheles* mosquitoes, migrate to the liver and replicate within hepatocytes to release merozoites. Merozoites replicate inside the erythrocytes in the bloodstream and release mature gametocytes which can, in turn, be taken up by the mosquito while feeding. Inside the mosquito’s midgut, the parasites undergo sexual development to form ookinetes, which penetrate the gut epithelium and develop into oocysts. These oocysts produce sporozoites that migrate to the mosquito’s salivary glands, completing the lifecycle [47].

Rodent malaria parasites such as *Plasmodium berghei* have been used as a model for human malaria research. Galizi et al. investigated whether specific tRNA cleavage occurs in *P*. *berghei* and found full length and 35 nt- fragments in Northern blots of total RNA extracted from *P. berghei* blood stages with probes specific to the 5′-end of tRNA^Gly_GCC^ [45]. In a later study, Wang et al. carried out deep sequencing of genome-wide small RNAs from *P. falciparum* and found that over 22% of the small RNA sequence reads mapped to tRNAs. Three species of tRNA fragments mapping to either the 5′- end, 3′- end, or the middle region of the parental tRNA were identified (they named these 5′ptRFs, mid-ptRFs and 3′ptRFs). 90% of these fragments were found to be derived from tRNAs encoding just eight specific amino acids confirming that their generation was not due to unspecific cleavage of all tRNAs [48]. However, the distribution of the different tRNFs varied amongst the above 8 tRNA genes. E.g. tRNA^Cys_GCA^ predominantly produced 5′tRFs, while tRNA^Ala_AGC^ produced lower amounts of 5′tRFs compared to the 3′- or mid- tRFs [48]. Interestingly, perceptible levels of 5′ tRNA^Ala_AGC^ have been reported in *trypanosomes* and *Entamoeba* and it appears that this particular tRNA half might have a role that is conserved across different parasites [13,14,30].

More recently, Hammam et al. showed that during nutritional stress (nutrient depletion is used to promote *Plasmodium* differentiation into gametocytes in research settings) tRNA^Asp_GTC^ was cleaved by an unknown nuclease, generating tRNA fragments [49]. tRNA fragments of *P. falciparum* origin were also found in the extracellular vesicles secreted by infected red blood cells (iRBCs). Interestingly, *Plasmodium* EVs were shown to deliver RNA cargo to human endothelial cells [50]. In a more recent study, tRNA fragments originating from *P. falciparum* were found to constitute over 96% of the parasite-derived small RNA cargo in the EVs isolated from iRBCs [51]. A majority of tRFs mapped to just four tRNAs- tRNA^Gly_GCC^, tRNA^His_GTG^, tRNA^Glu_CTC/TTC^, and tRNA^Pro_AGG^ similar to what was observed in Leishmania EVs [52]. The authors analyzed the tRF profile in the EVs produced from iRBCs subjected to either nutritional deprivation or amino acid starvation. Interestingly, a different set of tRFs were found to be selectively upregulated in these scenarios, which suggests a nuanced role for parasitic tRFs during stress survival/sensing [51]. However, the exact function of the tRNA fragments in these studies remains to be elucidated.

Table 1 highlights some recent research on tRNA fragments in the protozoan parasites.

## 10. Conclusions

Protozoan parasites overcome strong challenges as they invade the host organisms and need to rapidly adapt to stress and changes in the environment. tRNA derived fragments seem to provide a nuanced way to control gene regulation both within the parasites and outside- through transport in extracellular vesicles.

The mechanisms of biosynthesis and functional roles of various tRNA-derived fragments in parasite physiology remain an area of active research. Determining mechanism of cleavage of the parent tRNA molecules, including the RNases involved, and what triggers their activity will give important insight into their biology. Right now, the research has focused on a few, more abundant tRNA derived fragments. As more tRNA derived fragments and their functional roles, and mechanisms will be studied, a clearer picture should emerge about how various parasites use these molecules in their fight for survival. Future advancements, including AI-based deep learning, will allow for a deeper understanding of tRNA derived fragments by integrating the massive volumes of data being generated on sequence, structure, modifications, and binding partners.

## Figures and Tables

**Table 1:** Key findings and functions of tRNA fragments in various protozoan parasites.

Protozoan Parasite	Key Findings on tRNA-Derived Fragments (tsRNAs)	Functions/Roles Identified	References
***Entamoeba histolytica***	Various tRNA-derived fragments identified, mainly tRNA halves. Accumulation of tRNA halves under stress conditions like oxidative stress, heat shock, and serum starvation. tRNA fragments packaged into extracellular vesicles (EVs).	Modulation of gene expression during stress. Association with Argonaute proteins suggests regulatory roles. Intercellular communication via EVs.	[14]
***Giardia lamblia***	Discovery of stress-induced tRNAs (si-tRNAs) derived from the 3′ end of mature tRNAs. Accumulation during encystation and various stress conditions. Presence of tRNA-derived small RNAs (tRFs) involved in differentiation. tsRNAs found in EVs that interact with bacterial membranes.	Regulation during differentiation processes. Modulation of stress responses. Trans-kingdom communication affecting bacterial motility and biofilm formation.	[25,26,27,28]
***Trypanosoma cruzi***	Abundant 5′ tRNA halves, especially from specific tRNAs like tRNA^Asp_GUC^, tRNA^Glu_CUC^ and tRNA^Glu_UUC^ Upregulation during nutritional stress. Localization to cytoplasmic granules and association with PIWI proteins. tsRNAs present in EVs that can be taken up by mammalian cells.	Modulation of gene expression in host cells. Possible role in intercellular communication via EVs. Recruitment to stress granules during stress conditions.	[30,31]
***Trypanosoma brucei***	Abundant tRNA halves associated with ribosomes. Upregulation during stress conditions and stationary phase. Specific 3′ tRNA^Thr^ half stimulates translation during stress recovery. tRNAs undergo 3′ CCA tail shortening during stress.	Regulation of translation under stress. Enhancement of mitochondrial function during recovery. Facilitation of mRNA binding to ribosomes by tRNA halves.	[13,35,36]
***Trichomonas vaginalis***	Identification of tRFs and tRNA halves mapping to various regions of tRNAs. 5′ tRNA halves are the main type found in EVs. EVs are internalized by human cells.	Potential modulation of host gene expression via EVs. Intercellular communication between parasite and host cells.	[41,42]
***Leishmania* spp.**	tRFs derived from tRNAs like tRNA^Asp^, tRNA^Gln^, tRNA^Glu^, and tRNA^Leu^ found in EVs. Both 5′ and 3′ tRFs and tRNA halves identified. No direct correlation with amino acid usage.	Potential role in parasite gene regulation (not fully elucidated). Possible involvement in host-parasite interactions via EVs.	[42]
***Toxoplasma gondii***	Cleavage of tRNAs at anticodon loops yielding 5′ and 3′ tRNA halves. Enhanced tRNA half generation during stress and in avirulent strains. Higher amounts in quiescent stages compared to fast-growing stages.	Possible regulation of gene expression during stress and life cycle transitions. Role in metabolic adaptation and stage differentiation.	[45]
***Plasmodium* spp.**	Presence of tRNA fragments mapping to 5′, 3′, and middle regions of tRNAs. Specific tRFs upregulated during stress, e.g., cleavage of tRNA^Asp_GTC^ during nutritional stress. tRFs found in EVs secreted by infected red blood cells (iRBCs).	Potential modulation of host gene expression via EVs. Role in stress response and gametocyte differentiation. Intercellular communication affecting host-pathogen interactions.	[45,48,49,51]

## Data Availability

Not applicable.
